# Organic Winemaking and Its Subsets; Biodynamic, Natural, and Clean Wine in California

**DOI:** 10.3390/foods10010127

**Published:** 2021-01-08

**Authors:** Adeline Maykish, Robert Rex, Angelos K. Sikalidis

**Affiliations:** 1 Department of Food Science and Nutrition, California Polytechnic State University, San Luis Obispo, CA 93407, USA; amaykish@calpoly.edu; 2 Deerfield Ranch Winery, 10200 Sonoma Highway, Kenwood, CA 95452, USA; robert.rex@deerfieldranch.com

**Keywords:** oenos, organic wine, clean wine, biodynamic wine, sustainability, winemaking, natural wine, histamines, sulfites, wine-induced headache

## Abstract

From the ancient times, when wine/oenos was described as “Wine, the benevolent demon” by ancient Greek gastronomist and philosopher Athinaios in “Dipnosofistes”, to modern days, the craft has seen significant fruition. The wine industry has evolved over time, and more so recently, to encompass many different subsets, one of which is the organic wine market. The organic wine industry has grown in recent years, especially in California. This rapid gain in interest has resulted in the evolution of several subsets, including biodynamic, natural, and clean wine. While biodynamic and natural wine, function more as a fulfillment of niche markets, clean wine may provide benefits for consumers that otherwise suffer from side effects of wine consumption. Low sulfite levels and lack of histamines in clean wine plausibly decrease headaches and adverse effects some consumers experience when drinking wine. An overview of the organic wine industry and its evolution with potential contributions to consumers, with an emphasis on clean wine, is discussed herein.

## 1. Introduction

Organic winemaking as a concept has existed since the inception of organic farming, but unlike organic fruit and vegetable farming, it has struggled to gain market popularity and raise consumer awareness. Interestingly, it has not been until recently (2010s) that there has even been an organic wine market in the US. There are a variety of reasons for such delay, including a gradually increasing demand for artisan-made wines and a consumer base who wanted transparency in winemaking, with few to no added chemicals and maximum authenticity in the process [1]. In fact, in 2019, US sales of organic wine reached $80 million, a 28% increase since 2004 (https://www.organicconsumers.org/news/organic-wine-booming-across-us). While the market is still relatively small in terms of clientele and sales, it is growing as new types and branches of the organic wine industry develop [2].

**Figure 1:**
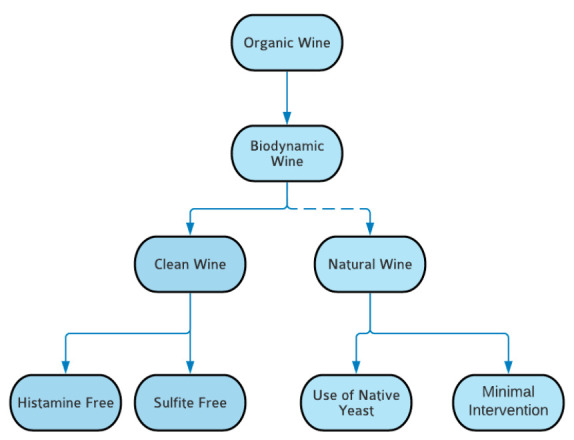
Illustration of the classification and flow of organic wine. Note: Natural wine is frequently implicated with biodynamic wine, but the two can be exclusive. This relationship is illustrated by the dashed line.

Consumer perception of organic wine continues to shift, as the market continues to change, and it becomes important to evaluate these ever-shifting views. In a 2019 study investigating the willingness to pay (WTP) more for organic wine, 500 Italian wine consumers were randomly surveyed. Characteristics regarding general wine consumption, organic wine consumption patterns, intrinsic and extrinsic characteristics of organic wine purchase, WTP, and sociodemographic characteristics were all collected. In total, 57% of participants were male, while 20% of participants were 18–35 years old, 41% 36–50, 33% were between 51–65, and the remanding 5% were over 66. Overall, 73.2% of consumers reported an unwillingness to pay more, but this was determined to be due to a lack of means to do so, not because of a lack of interest or persuasion. Of participants who did have the means to pay a higher price, 13.2% reported being willing to pay 20% more. Therefore, if an organic winemaker wanted to market their wine as “premium”, the most ideal pricing would be 20% more than a standard, nonorganic bottle of wine. Participants also reported the increased WTP due to environmental concerns and the distinctness of organic wine, illustrating increased interest in sustainability that consumers now hold [3]. This constitutes an example of the shifting market—at its inception, while organic farming was heavily focused on generating a “better-for-you” product, over time, interest has seemed to be shifting to also include more sustainable practices and environmental concerns, generating new subsets of organic wine (Figure 1). However, the sector can be clouded by lack of definition and standardized regulation. The aim of this review was to discuss the organic wine market and its subsets to clarify the similarities and differences among the sectors.

## 2. Food Safety and Winemaking

The Food Safety Modernization Act (FSMA) was created in 2011 out of a drive to increase precautions and ensure food safety in the United States, and it was the first significant piece of legislation to address food safety since 1938 [4]. It has become the standard for all food processing across the United States, aiming to ensure quality and safety in food products, including wine. Wine has been long regarded for its history of safety and lack of bacteria due to fermentation, but is still included in FSMA [5]. FSMA states that every winery will be visited at least once within the first seven years of its enactment, and then regularly thereafter [6]. Wineries that generate <$500,000/year with at least 51% of income from food sale direct to consumers or local retailers, or wineries that qualify as a “very small business” are required to maintain systematic records to ensure proper practices and notify the Food and Drug Administration (FDA) of compliance with regulations. All other wineries must comply with a formalized education and training program for employees; proper recording of current good manufacturing practices, sanitary facilities, and controls; and must record each ingredient used in each wine [7,8]. No nutrition or ingredient labeling is currently required, although that cannot be excluded in the future [8].

There has been some concern regarding safety in organic wine, especially considering the ban on pesticides and the potential introduction of foodborne illness caused by animal exposure in outdoor conditions [9]. However, it has been supported that safety is, in fact, greater in organic foods as related to metabolic and long-term health parameters, due to lower nitrogen applications, the ban on pesticides, and the ban on prophylactics, all of which may decrease cancer risk in consumers [9]. While there is still potential for exposure to foodborne pathogens, risks can be mitigated through increased safety measures during processing to ensure high quality, thus minimizing actual food safety risk [9].

Ochratoxin A (OTA) poses a significant risk in winemaking due to its nephrotoxic, mutagenic, and teratogenic effects at high concentrations [10]. Gentile et al. investigated OTA presence in organic wine, a toxin that, when present in high concentrations, can produce nephrotoxic, mutagenic, and teratogenic effects. Fifty-five different wine samples (*n* = 40 red, *n* = 15 white) during two vintages were assessed, and toxin detection was done using ultra performance liquid chromatography (UPLC). OTA presence is allowed in wine at a maximum concentration of 2 ng/mL. It was found that the red wine samples had a higher presence of OTA than white, but all samples had an OTA level below 1.0 ng/mL. In total, 53.3% of the white wine samples contained OTA levels below the limit of quantification, and all samples demonstrated OTA levels below 0.1 ng/mL [10]. When compared to commercial wines, it was observed that organic winemaking does not increase OTA incidence [10].

It constitutes standard practice for wineries to select pure yeast strains in order to control fermentation more effectively and ensure quality [11]. Prior to fermentation, bacteria are present, but as fermentation enters its final stages, most bacteria are killed-off due to the alcohol content rising levels and the low pH value. Therefore, regardless of wine production type (organic versus nonorganic), it is the fermentation step that is vital to ensuring safety in the final product, thus rendering both organic and nonorganic wines similar in terms of food safety for the consumer [11].

## 3. Wine Microbiology and Chemistry

Wine microbiology and wine chemistry are crucial components to winemaking, and extra attention to these characteristics is typically placed in organic winemaking, making them vital to ensure high quality and a unique product. While organic wine is relatively similar biologically to conventional wine, there are some key differences distinguishing the two, such as yeast presence and chemical composition, thus somewhat differentiating the microbiology and chemistry of organic wines compared to conventional ones.

Perhaps the biggest difference between organic and conventional wines are the yeast strains typically present in each. This difference in the demography of yeast can then affect sensory attributes after fermentation, potentially creating a distinct difference between organic and conventional wine. Xu et al. investigated yeast succession during spontaneous fermentation of grapes from both conventional and organic vineyards. Wine was fermented using standard fermentation practices and yeast was identified using high-throughput sequencing. A group of 18 total yeast genera and 36 species of yeast were identified, with *Saccharomyces*, *Hanseniaspora*, and *Ascomycota* being the most abundant. Overall, organic grapes exhibited higher occurrence of *Saccharomyces*, which dominated during fermentation. The overall diversity of organic wine decreased more dramatically during fermentation than conventional wine as well. Sensory analysis was also conducted, with conventional wines presenting more acidity and sweetness, whereas organic wines scored better in terms of clarity, alcohol, and fruitiness, as assessed by the panel. The organic wine was also determined to have a mellower mouthfeel. The differences in flavor and the aroma are attributed to the yeast strain and understanding the diversity of yeast in organic wine may constitute a predictor for quality and overall flavor, while also informing the aroma and the bouquet of the wine [12].

Spontaneous fermentation typically utilizes non-*Saccharomyces* yeasts for ethanol production, which results in higher yeast diversity, increased aroma, and significant and clearly noticeable differences in flavor profile compared to conventional wine [13]. Some non-*Saccharomyces* yeasts are described often as “killer” yeasts, with the ability to inhibit spoilage yeasts. Combined use of these (“killer”) yeasts can result in optimal flavor profiles with decreased risk for spoilage. Benito et al. investigated the concept while they fermented Tempranillo grapes with a culture of *Schizosaccharomyces pombe* and *Lachancea thermotolerans* to undergo malolactic fermentation. Typical malolactic fermentation can result in off-flavors and other quality issues. However, through the approach the researchers employed, *Schizosaccharomyces pombe* stabilized the wine, as it evidently consumed all malic acid initially present. *Lachancea thermotolerans* then produced lactic acid, finalizing the process. As a result, risk of quality issues related to type of yeast was diminished, and the wines produced demonstrated lower levels of acetic acid and biogenic amines. While this set of experiments addressed merely one use of “killer” yeasts, it does hint towards opportunity in the wine industry to improve wine quality through yeast strain selection [14]. Both organic and conventional winemaking can utilize spontaneous fermentation, but as illustrated in the previous study [13], organic wine made through this process can also result in higher yeast diversity. This can effectively create an opportunity in the organic wine market to utilize these yeasts, as the wine may be significantly better equipped to handle these yeasts during fermentation.

In a separate set of experiments, Setati et al. utilized the theory of sampling to sample grapes from three adjacent vineyards in South Africa for a better understanding of the vineyard microbiome. The three vineyards differed regarding their agricultural practices, while they were also under different management. Out of the three vineyards used, one was conventional, the second biodynamic, and the third integrated in terms of production practices. Integrated production can be defined as an agricultural approach that is more environmentally friendly, but is not regulated by a particular system or certification body. The authors reported that the total yeast population was higher in both the biodynamic and conventional vineyard than the integrated, and total population ranged between 4–8 × 104 CFU/g for all vineyards. The biodynamic vineyard displayed the most unique biodiversity, with the identification of several yeasts identified as responsible for biocontrol. All three vineyards exhibited high levels of oxidative yeasts; however, these yeasts cannot survive in winemaking, and therefore do not affect quality ultimately. While the biodynamic vineyard did demonstrate the most species richness, vineyards are subject to fluctuation, which may be responsible for the differences in intra-vineyard wine quality. However, vineyard mapping is still of importance, especially when considering biodynamic wine, in order to gain a better and a more inclusive understanding of the vineyard footprint, while also to better comprehend how growing conditions may impact wine flavors [15].

Organic and conventional wines have also indicated differences in the chemical composition level. Polyphenol oxidase (PPO) contributes to browning and is present in grapes. The PPO levels in organic and conventional grapes were assessed, with grapes from three sections of each vineyard [15]. It was found that PPO activity in the organic grapes was twice that of conventional, which could be a result in changes in the phenolic metabolism when grown with or without synthetic chemical pesticides. The high PPO activity may also contribute to disease resistance, as PPO allows for the rapid oxidation of phenols to quinines, effectively inhibiting polygalacturonase, an enzyme responsible for the degradation of the cell wall [16]. A brief summary of the various characteristics is provided on Table 1.

Mulero et al. investigated antioxidant activity and phenolic compound presence in organic red wine using three different vinification processes. *Monastrell* grapes were used, all of which were grown organically. Grapes underwent either vinification after prolonged maceration (21 days), vinification with the addition of enological enzymes, or traditional vinification procedures (10-day maceration). Additionally, 70 mg/kg of SO_2_ was added. Total phenolic compounds and antioxidant activity were then measured. It was found there was no significant difference in antioxidant activity among the three vinification processes. The average total phenolic compounds in wines made with the addition of enzymes was not significantly different than those made with traditional methods. Total phenolic compounds were initially higher in wines with a prolonged maceration time but decreased after three months of storage. Overall, there was no significant difference between the three processes and phenolic compound concentration. While all three of these processes utilized the same organically grown grapes, the results are still of interest. It is likely that the grapes themselves contribute to phenolic compound concentration, not necessarily the growing practices [17].

Vilanova et al. studied aromatic compound presence in three red cultivars—Caiño Tinto, Caiño Longo, and Caiño Bravo. Two vintages were studied (2002 and 2003). Grapes were crushed by hand and allowed to undergo spontaneous fermentation for 14 days. It was not mentioned whether grapes were organically or conventionally grown. Alcohol, ester, and acetate presence was determined by gas chromatography. 3-methyl-butanol was the most common alcohol, though the concertation for the three cultivars varied significantly. In 2002, Caño Bravo produced the highest concentration of higher alcohols, whereas in 2003, Caño Tinto produced the highest. Ester and acetate contents were significantly different among the wines as well, with the majority of esters being found in the Caño Longo wines. Ethyl acetate was the most commonly seen ester. Overall, the 2002 vintage was more similar across the three cultivars than the 2003 vintage [18]. This again illustrates that it is more likely the grape, rather than the growing practice, that contributes to flavor and chemical makeup of the wine.

It is also worth noting that there is a knowledge gap in terms of chemical makeup of organic versus conventional wine, with research focusing on just one of the two. This may be due to the likelihood that the grape is the major contributor to differences, but may be worth further investigation.

It becomes evident that organic wine significantly differs from conventional wine biologically. However, there is also variation within the subset. Parpinello et al. compared Sangiovese wines from organic and biodynamic vineyards, looking at both the differences in chemical composition and sensory differences. Grapes from two consecutive vintages (2011 and 2012) from both organic and biodynamic vineyards were harvested, and wine was made following European Union regulations (European Food Safety Authority, EFSA). Result sets of chemical analyses were then compared to one another, as well as to those obtained from a previous study that investigated the chemical analysis of Sangiovese wines in a vineyard that was converted from organic to biodynamic viticulture (2009 and 2010 vintages) [19]. Both the 2011 and 2012 vintages behaved similarly, with no significant difference in color intensity and total polyphenols. When compared to the previous vintages however, the 2011 and 2012 vintages showed significantly decreased differences in volatile components. In terms of sensory conditions, the 2011 biodynamic vintage was reported to be fruitier, whereas acid was higher in the organic wine. Consumers did not show a clear preference for one wine over the other. It was thus evident that transition from organic to biodynamic farming may cause changes in chemical and sensory attributes during transition, but these quickly return to pretransition attributes. It also became evident from the study of Parpinello et al. that no major chemical differences were observed when comparing biodynamic to organic wines. However, it is important to note that only one wine grape was studied, and results are not inclusive of every type of biodynamic winery [20].

## 4. Biodynamic Wine

One interesting subset of organic wine is biodynamic wine. Biodynamic wine is loosely defined as a step further beyond organic. The general concept is that everything in winemaking is interconnected, and there must be a balance between the characteristics of the vine used, the means and labor practices, and the characteristics and treatment of the soil for an overarching holistic approach to winemaking [21]. Biodynamic winemaking primarily stems from an increased interest in sustainability, with an emphasis on generating a self-sustaining eco-friendly system [20,21]. Although the term has been present since the 1920s, it was not until the late 1990s that it became more commonly used [21,22].

The primary difference between organic and biodynamic wines in terms of labeling is the levels of sulfites allowed at bottling. Typically, organic farming and biodynamic farming are closely related and coexist, hence it is not uncommon to see both on the label. Certified biodynamic wines can contain up to 100 ppm of sulfites, whereas organic wine must contain less than 10 ppm naturally occurring sulfites [23]. However, biodynamic winemaking may be more sustainable than organic, as it does not allow for the use of the herbicide “Roundup” [24]. A 2014 study investigated the environmental impact of biodynamic, conventional, and biodynamic-conventional (biodynamic without official certification) winemaking using life cycle analysis (LCA) [25]. All vineyards were in Spain, and a total of 45.6 hectares were evaluated. It was found that certified biodynamic winemaking produced the lowest environmental burdens, whereas conventional winemaking produced the highest. This was due to an 80% decrease in diesel inputs in biodynamic winemaking, minimal use of plant protection products and fertilizers, and use of manual labor instead of mechanical [25]. While biodynamic-conventional, not organic, was the third practice investigated here, it is closely related to organic. This study illustrates the potential for biodynamic wine to be the most sustainable and should be of interest for individuals with an interest in sustainability.

## 5. Natural Wine

The term “natural wine” does not inherently mean the wine is made biodynamically, however the two are frequently implicated [26,27], and is therefore worth discussing herein. Natural wine lacks a strict definition as well, but refers to winemaking free of added pesticides, chemicals, and other additives [28,29]. It requires the use of organic farming, indigenous yeast, no additives, minimal intervention during fermentation, and low final sulfite concentrations [29] and is frequently described as “low intervention” [30]. Natural wines are also “un-fined” and unfiltered, meaning that any microbes or proteins in the wine remain present in the bottle [31]. The resulting product tends to be slightly cloudy/turbid, with “funky”, less fruity tasting notes. The use of indigenous yeast may increase histamine levels [31]. This cloudiness surrounding the term is likely due to the novelty of the market. While it can be argued that the first wines created were indeed “natural wines”, modernization of the market led to increased intervention and additives to create a more desirable product. The natural wine movement is believed to have begun in France in the 1960s, with the goal of creating a product without pesticide use and addition of chemicals [32]. However, the industry truly began to boom after Isabelle Legeron popularized the product in 2009 with her Raw Wine [33]. The novelty of the product garnered significant attention, causing increased interest in the natural wine movement [27].

The recent passing of a natural wine certification looks to clarify the market. In March 2020, the Institut National de l’Origine et de la Qualité (INAO), located in France, recognized a definition of the wine proposed by the Syndicat de Defense des Vins Naturels. This certification broke the wines down into two levels: wines made with no added sulfites and wines that use less than 30 mg/L of sulfites [34]. However, this definition still remains problematic, as some yeasts can produce more than 30 mL/L of sulfites during fermentation, making some natural wines ineligible for certification [34]. This certification is only available for French wine as well, resulting in the continued confusion and muddled definition around the world [35].

Due to the increasing market for natural products overall, natural wine has garnered considerable interest rather quickly. A study in Italy involving 286 wine consumers investigated WTP for natural wine, to further understand the effects of labeling and the thought and decision-making process of consumers behind choosing natural wine. On average, research revealed that participants were willing to spend €7.32 (±€1.69), €2.32 more than a conventional bottle of wine. The WTP increased due to interest, drink occasion, label information on ingredients, organic production method, sensory characteristics, vintage year, and income, indicative of an overall high level of interest in natural wine. Millennial versus elder WTP showed a decrease, meaning millennials were more willing to pay for natural wine compared to older generation representatives [28]. This is likely due to an increased interest in organic and natural products among millennial consumers. While this study focused primarily on Italian wine consumers, it illustrates an overall interest in natural wine, especially among younger wine consumers plausibly expected more widely.

## 6. Clean Wine

For the purpose of this article, an emphasis will be placed on clean wine, as it is the least defined of the three categories and often presents significant controversy. Clean wine as a category, is a subset of organic and biodynamic wine and extends further than the use of organic wine grapes. While there is sometimes the misperception that “clean wine” in nonalcoholic, that is not the case according to the trademark as approved by United States Patent and Trademark Office (USPTO). Little evidence surrounding clean wine is published, due to the fact that it is a very new topic and carries rather limited formal definition. Clean Wine^®^ has been trademarked by Deerfield Ranch Winery (DRW), whereby: “wine is made cleanly from start to finish to generate a sustainably made product free of sulfites and histamines” [24]. Due to the fact that the concept is trademarked in California and the US, Clean Wine^®^ will be defined using Deerfield Ranch Winery’s definition officially approved by the US Patent and Trademark Office, as it is the most well characterized and established one officially available. The clean wine approach aims to effectively create a product that otherwise sensitive individuals can enjoy. Clean wine is grown organically and biodynamically, and after harvest the grapes are triple-hand sorted, handled, and processed in a clean facility to minimize grape stress, resulting in less bacterial competition and minimizing the need for additives. Clean wine and natural wine both aim to decrease additive use, either by using less or removing them from the equation completely. However, the two do so for different reasons. Natural winemakers look to do so to return winemaking to its roots and minimize intervention. Clean winemakers decrease additive use for health reasons, above all else (raw wine source, Deerfield source).

It is important to note that headaches commonly associated with wine consumption are a complex and multivariable issue. Body mass, time, gender, age and genetics all play a role in alcohol metabolism, and effectively headache onset [36,37,38]. As with any alcohol, wine is dehydrating. If the consumer is not well hydrated, chance of headache is increased due to these dehydrating effects [39]. Similarly, the type of food consumed before or while drinking can also have an effect since foods high in sodium will further dehydrate the body, again contributing to headache. It is recommended to consume protein and nutrient-rich fruit and vegetables prior to alcohol consumption to stabilize [40]. In fact, food consumption prior to drinking has been linked to increased alcohol elimination [41], when compared to those in a fasting state. Given alcohol is absorbed at the level of the stomach, consumption of lipid also helps with slower uptake of alcohol, thus allowing more time for alcohol dehydrogenase and the overall alcohol metabolism to occur optimally, thus minimizing undesirable side effects post drinking.

Alcohol metabolism harbors a significant genetic factor in terms of the alcohol dehydrogenase enzyme (ADH). The several alleles of ADH have been attributed to either increased or decreased alcohol metabolism, or the rate at which alcohol is converted to acetaldehyde [42]. It is evident that it is not just histamine or sulfite presence in wine that causes headaches, but many other factors as well. However, histamine and sulfite presence tend to be considered less when discussing effects of alcohol consumption, leading to clean wine production.

### 6.1. Sulfites

In order to discuss clean winemaking, it is important to consider the fermentation science that results in histamine and sulfite production. Sulfite-free wine is a relatively new concept, crafted from misconception and misinformation. In fact, it was not until the 1980s that sulfites in wine even became an issue discussed from a consumer’s standpoint in the US. An FDA-commissioned study found that 1% of the population could be sulfite sensitive, and therefore enacted mandatory labeling of sulfite content in wines [43]. While winemakers are able to make low-sulfite wines, it is virtually impossible for wines to be actually sulfite-free.

Sulfur dioxide (SO_2_) is a vital agent to winemaking, since without the addition of SO_2_, grapes would ferment to a point that would render the wine undrinkable. Furthermore, SO_2_ also functions as a preservative, preventing oxidation and excess bacterial growth from occurring, hence making wine safer. Winemakers typically add SO_2_ during grape maceration to help prevent mold and spoilage. It depends on the winemaker’s discretion as to under what conditions to add more SO_2_ and how much. Depending on the school of thought and approach to the craft, winemakers in California are typically conservative, adding as little as possible—just enough to keep the wine “wholesome” during fermentation. By the time the wine is bottled, it contains less than 10 ppm total SO_2_, and therefore does not have to be labeled as “Contains Sulfites” by FDA regulations [44], regardless of the fact that it may actually contain low levels of sulfites. While there are other processes for preservation, such as pasteurization, SO_2_ addition is the most effective. It is also deeply rooted in the winemaking history and practice since the first documented use of sulfur dates back to 1487 [45].

There is certainly consumer interest in sulfite-free wine as well. In a 2014 study, a panel of 223 individuals participated in a survey investigating the perception of sulfites in wine. It was found that 34% of those surveyed reported headaches after consuming red wine, which was at the time attributed to sulfites. It was found that those surveyed reported a WTP of $1.23 more per bottle to avoid sulfites. Participants were also 3.4% more likely to purchase a sulfite-free wine. However, consumers still ranked value, quality, and taste of more concern than sulfite presence, meaning that sulfite-free wine must still perform in terms of taste and price to be competitive in the market [43]. While it is now known that sulfite insensitivity can cause allergic reactions (not headaches), the aforementioned data are still rather interesting. Consumer interest in learning more about what is in wine, a WTP for a higher price for cleaner wine, and the confusion that is typically associated with the wine market are all demonstrated. Minimizing the source of adverse reactions to drinking wine and investigating consumer interest is key for development of a clean wine market.

In another study conducted in Sicily, 201 individuals participated in face-to-face surveys regarding sulfite-free wine and interest surrounding the product. It was found that women were 3.8% more likely to pay 10% more for a sulfite-free wine but were 34.7% less likely to pay a premium price for the same product, expressing a very specific price point that sulfite-free winemakers would need to target. It was also found that curiosity was the primary reason behind those surveyed willing to pay more, and environmental concern was also a significant reason behind a willingness to pay more. It is important to note that a majority of those surveyed were in the 41–60 years old age range (105 individuals) [46], meaning that these results may be indicative of a more characteristic age group rather than the general population. Curiosity and environmental concerns may be greater for a younger group than an older group, whereas premium price may be of more interest to an older population [46].

### 6.2. Histamines

Sulfites were long considered to be the source of headaches in red wine [47]. However, when individuals still experienced severe headaches and reactions to sulfite-free red wine, further questions were raised regarding the association between post drinking sessions headaches and sulfite content of the wine. Further studies determined that histamines, rather than sulfites, in red wine could be a more likely cause for headaches, due to the fact that red wine is fermented with the grape skin on. Histamines present in wine are derived from the grape skin, providing the potential for adverse reaction [47].

Histamine intolerance is the result of excess histamines in the body and lack of enzyme production/function to degrade it. Diamine oxidase (DAO) is the enzyme responsible for histamine degradation, and when enzyme activity is reduced, individuals may experience more challenges with metabolizing high histamine containing foods and beverages such as cheese, smoked meats, and wine [48].

In a study aimed at investigating the role of histamine in red wine intolerance, 38 individuals participated in a tasting session. Overall, 28 individuals reported previous wine intolerance and 10 reported no wine intolerance whatsoever. All participating individuals tasted 125 mL of red wine with approximately 50 μg histamine. Plasma histamine and lung function were assessed before, at 15 min after consumption, and at 30 min after consumption. In total, 22 individuals demonstrated significantly higher plasma histamine levels after 30 min, in comparison to the control group, and two experienced a mild asthma attack. Histamine levels in red wines were then assessed, and levels ranged from 60–3800 μg/L [49]. Due to the reaction seen at 50 μg, it is likely that histamines in red wines are causing wine intolerance, and as little as 50 μg can still cause a reaction, although the intensity of which may not be easily predicted.

In a study investigating DAO deficiency and histamine degradation, 45 individuals participated in a four-week-long dietary study. Participants were placed in one of two categories: (1) chronic headaches from eating certain foods (*n* = 28), or (2) intolerance to various foods/wine (*n* = 17). They were asked to eliminate fish, cheese, cured sausages, pickled cabbage, alcoholic beverages, and other high histamine foods from their diets. After a four-week period, 33/45 participants reported over a 50% reduction in symptoms (19/28 in group 1, 14/17 in group 2). They were then asked to consume histamine rich foods, and symptoms were found reproduced. These results indicated that histamines were associated with headaches and intolerance, while researchers attributed this to a potential deficiency of DAO [50].

While histamines are introduced to the wine through the grape skin, it is the interactions during fermentation that result in a potentially high histamine level in wine. Bacteria on the grape surface, within damaged grapes, and in any other grape material (leaves, branches, etc.) compete with yeast during fermentation, and when bacterial counts become excessive, high levels of histamine formation occurs. It has been determined that lactic acid bacteria (LAB) are the typical source of histamine formation, as they contain histidine decarboxylase (HDC). HDC is responsible for converting histidine to histamine [51]. In order to streamline red wine production and reduce histamine formation, 136 LAB were analyzed to determine which strain produced the highest histamine levels. It was found that *Oenococcus* produced the lowest concentrations of histamines, whereas *Pediococcus parvulus* and *Lactobacillus hilgardii* produced the highest. The latter two are spoilage organisms, and cause other adverse reactions during fermentation as well, which therefore renders it vital to minimize bacterial growth [52]. While smaller wineries may not be able to run large scale bacterial testing, it may be beneficial to run microbiological sampling to determine baseline bacterial presence.

As previously stated, bacteria are introduced in the fermentation process during harvest. It is therefore vital to thoroughly sort grapes to remove fruit that may be damaged or spoiled and to remove any leaves or branches as well as other materials that may become mixed in. In doing this, winemakers significantly reduce bacterial levels at baseline, and hence typically start with a “cleaner” product from the beginning. Before fermentation initiates, it is vital to thoroughly sanitize all surfaces and equipment, while also follow good fermentation science practices, again to minimize bacteria levels. When effectively doing so, yeast strains are presented with less of a bacterial load to compete with, and as a consequence, yeast die-off is significantly reduced. This condition also results in a cleaner wine, as histamine levels are much lower in wines crafted through this process. Careful monitoring of yeast during fermentation also helps prevent die-off, again reducing histamine presence leading to an overall higher quality wine [24].

## 7. Organic Winemaking and Sustainability

While organic agriculture does not inherently equal sustainable, it can be argued that sustainable agriculture became more popular due to organic agriculture. It is important to distinguish the two—while organic agriculture is defined by crop rotation to maintain nutrients in soil, no use of pesticides, planting of organic seeds, and growth of food free of genetic engineering, sustainable farming is more focused on the land and processes that enhance natural resources such as water, enhance life for farmers and those consuming the product, utilization of resources in an efficient way, and agriculture that is economically viable [53]. From the descriptions, it becomes evident that organic agriculture serves as a stepping stool to sustainable agriculture.

The subsets discussed herein all stem from a desire to increase sustainable practices in the winemaking industry. It takes approximately 1.2 kg of grapes to produce a standard bottle of wine, and a significant portion of this weight does not transpire in the bottle. This effectively creates a significant amount of pomace (grape skins, seeds, and stems) that may potentially go to waste. Between 1.3 to 1.5 kg of waste is generated per liter of produced wine available for consumption, while 75% of that waste is attributed to wastewater [54].

While winemaking typically is regarded as a relatively environmentally friendly process, it does still generate significant amounts of waste, leaving room for innovation in terms of waste management and utilization. Research involving repurposing of grape waste has been of interest, particularly due to the fact that this pomace is rich in antioxidants that can prevent biological damage [48]. Pomace can be used as fertilizer; to make a second, weaker wine; or in dietary supplements and cosmetics [55]. Seed oil and extracts are also frequently made from pomace [56]. Wine grape juice extract is used for the development of premium nonalcoholic wine beverages [57]. There is a steady trend towards more sophisticated food and beverages with a variety of considerations for the consumers spanning from ethical and fair trade to sustainability and health benefits [58].

## 8. Conclusions

The organic wine sector contains many niche markets but struggles from one overarching theme—lack of clear definition. This can be difficult for a skilled winemaker to decipher, never mind the average consumer, due to overlap and lack of universal regulation (Table 2). American wine can be labeled as “made from organic grapes” in America but can be sold as “organic wine” in Europe, whereas European organic wines must include “made from organic grapes” to be sold in America, providing just one example of the lack of congruency among the market [59,60]. This effectively creates a confusing market for consumers as well, generating a lack of trust surrounding labels and a decreased WTP a premium price [61]. Distinct universal clarification of organic, biodynamic, natural, and clean wine are all necessary to strengthen the market and establish standards for the industry. In doing so, a larger consumer base may be generated, as education and clarity surrounding the product may increase, garnering larger interest. Novel products involving premium wine and wine grapes could thus also be developed, while also their market penetration facilitated [62,63].

## Figures and Tables

**Table 1:** Summary of chemical and microbial characteristics in conventional vs. organic wine. Strength of differences is illustrated by +/−, i.e., one + demonstrates a probable difference, whereas 2 demonstrates a stronger difference.

Wine Type	Biodiversity	Yeast Strains	PPO Presence	Sensory Attributes	Disease Resistance	References
Conventional	− −	*Hanseniaspora*	− −	Acidic, sweeter	−	[12,13,14,15,16,17,18]
Organic	+ +	*Saccharomyces*	+ +	Mellow mouthfeel, increased clarity, increased fruitiness	+	[12,13,14,15,16,17,18]

**Table 2:** Summary of general similarities and differences of wines in the organic wine sector.

Wine type	Established Regulations/Certifications	Agricultural Regulations	Additives
Biodynamic	Yes—United States	No use of roundup, sourcing of materials from winery	Up to 100 ppm sulfites
Natural	Yes—France	No added pesticides, typical overlap with biodynamic	Less than 30 mL/L sulfites, no other additives
Clean	None	Typical overlap with biodynamic	Less than 30 mL/L sulfites

## Data Availability

The data presented in this study are available on request from the corresponding author.
